# TM4SF5-mediated liver malignancy involves NK cell exhaustion-like phenotypes

**DOI:** 10.1007/s00018-021-04051-x

**Published:** 2021-12-18

**Authors:** Hyunseung Sun, Eunmi Kim, Jihye Ryu, Hyejin Lee, Eun-Ae Shin, Minhyeong Lee, Haesong Lee, Jeong-Hoon Lee, Jung-Hwan Yoon, Dae-Geun Song, Semi Kim, Jung Weon Lee

**Affiliations:** 1 Department of Pharmacy, College of Pharmacy, Seoul National University, Seoul, 08826 Republic of Korea; 2 Research Institute of Pharmaceutical Sciences, College of Pharmacy, Seoul National University, Seoul, 08826 Republic of Korea; 3 Department of Internal Medicine and Liver Research Institute, Seoul National University College of Medicine, Seoul, 03080 Republic of Korea; 4 Natural Product Informatics Research Center, Korea Institute of Science and Technology (KIST), Gangneung-si, Gangwon-do 25451 Republic of Korea; 5 Immunotherapy Convergence Research Center, Korea Research Institute of Bioscience and Biotechnology, Daejon, 34141 Republic of Korea

**Keywords:** Immune checkpoint, NK cell immune therapy, Liver cancer, Signal transduction, L6 family member

## Abstract

Aberrant extracellular matrix and immune cell alterations within the tumor microenvironment promote the pathological progression of liver carcinogenesis. Although transmembrane 4 L six family member 5 (TM4SF5) is involved in liver fibrosis and cancer, its mechanism avoiding immune surveillance during carcinogenesis remains unknown. We investigated how TM4SF5-mediated signaling caused immune evasion using in vitro primary cells and in vivo liver tissues from genetic or chemically induced mouse models. *TM4SF5*-transgenic and diethylnitrosamine (DEN)-induced liver cancer mouse models exhibited fibrotic and cancerous livers, respectively, with enhanced TM4SF5, pY^705^STAT3, collagen I, and laminin γ2 levels. These TM4SF5-mediated effects were abolished by TM4SF5 inhibitor, 4′-(*p*-toluenesulfonylamido)-4-hydroxychalcone (TSAHC). TM4SF5-dependent tumorigenesis involved natural killer (NK) cell exhaustion-like phenotypes including the reduction of NK cell number or function, which were blocked with TSAHC treatment. TM4SF5 expression in cancer cells downregulated stimulatory ligands and receptors for NK cell cytotoxicity, including SLAMF6, SLAMF7, MICA/B, and others. TM4SF5 suppression or inhibition reduced STAT3 signaling activity and recovered the receptor levels and NK cell surveillance, leading to reduced fibrotic and cancerous phenotypes, and longer survival. Altogether, these findings suggest that TM4SF5-mediated STAT3 activity for extracellular matrix modulation is involved in the progression of liver disease to HCC and that TM4SF5 appears to suppress NK cells during liver carcinogenesis.

## Introduction

Chronic liver injury progressively causes liver diseases, including nonalcoholic fatty liver disease (NAFLD), via excessive production of the extracellular matrix (ECM), which drives fibrosis and cirrhosis and ultimately results in hepatocellular carcinoma (HCC) [1]. Specifically, the excessive ECM deposition during fibrosis and cirrhosis activates abnormal cell proliferation signaling for HCC development [2].

Nonalcoholic injury-mediated liver inflammation results in the production of diverse cytokines, which can facilitate ECM production for preneoplastic progression and eventual carcinogenesis. In addition to the influence of the inflammatory environment, immune cells in the microenvironment are critically associated with the carcinogenic processes [3]. After their transformation from normal to malignant cells, cancer cells present tumor antigens to T cells, and antigen-presenting cells (APS, including dendritic cells, B cells, and macrophages) play a crucial role as nexus, performing cross-presentation of tumor-derived antigens. The identification of cancer-associated antigens by APCs then leads to T-cell priming and tumor infiltration, which eventually leads to T-cell-mediated recognition and killing of the tumor cells [3]. During this process, immune checkpoints, such as cytotoxic T lymphocytic protein 4 (CTLA-4) and programmed cell death protein 1 (PD-1), on immune cell surfaces have been actively targeted to boost the immune system and for anti-tumor immunotherapies [4]. Recently, the anti-cancer efficacy of antibodies against PD-1/programmed cell death ligand 1 (PD-L1) via T-cell checkpoint blockade in obese cancer patients [5] and non-small cell lung cancer [6]. Despite significant immune surveillance in the liver, HCC can still occur as a result of chronic liver infection or inflammation. Natural killer (NK) cells make up approximately 50% of the liver lymphocyte population and kill tumors via the release of pro-inflammatory cytokine interferon-γ (IFN-γ) [7] and cytotoxic granules, such as perforin and granzyme [8], as well as via death receptor signaling (TNF-related apoptosis-inducing ligand [TRAIL] and Fas Ligand [FasL]) [9]. Unlike cytotoxic CD8^+^ T-cells, NK cells are activated through a cascade of various activation/inactivation receptors on their cell surfaces, rather than by antigen presentation [10]. NK cell-mediated cytotoxicity relies on “missing-self” and “induced-self” recognition to identify target cancer cells by maintaining a precise balance between co-stimulatory and co-inhibitory signals via functional receptors. These interacting signals eventually lead to the activation and functional status of NK cells [11]. Because the liver provides a robustly immunosuppressive microenvironment compared to other organs and contains a large population of NK cells that express immune checkpoints, it is the least responsive organ to current checkpoint immunotherapies in patients with liver cancer and metastases [12]. The high frequency of liver-resident NK cells in the healthy liver also suggests an essential role of NK cells in targeting and preventing liver metastasis. Although we currently have a good understanding of the complex and often redundant activation/inactivation pathways of NK cells [13], HCC-specific checkpoints in hepatocytes and NK cells warrant further investigation.

Transmembrane 4 L six family member 5 (TM4SF5, L6H also as a member of L6 four transmembrane superfamily [14]) is an *N-*glycosylated membrane protein with four transmembrane domains [15]. It is induced by transforming growth factor β (TGFβ1) signaling in a carbon tetrachloride (CCl_4_)-induced liver fibrosis animal model [16] and is also highly expressed in clinical human liver cancer tissues [17], in addition to increased expression in colon, prostate, gastric, pancreatic, and esophageal cancers [18, 19]. Macrophages, hepatic stellate cells, and endothelial cells in liver also show to express TM4SF5, with being increased upon inflammatory environment (data not shown). TM4SF5 activates non-receptor tyrosine kinase c-SRC via direct physical association [20], which leads to signal transducer and activator of transcription 3 (STAT3) activation [21]. Although TM4SF5 is involved in animal liver fibrosis and xenograft growth, the mechanism by which TM4SF5 is influenced by the immune system, particularly via NK cells, during hepatic carcinogenesis remains unknown.

In this study, the roles of TM4SF5 in the development of precancerous and cancerous phenotypes in genetically engineered or chemically treated animals were explored in regard to TM4SF5-dependent signaling and crosstalk between cancer cells and immune cells. We found that TM4SF5-mediated STAT3 activity caused excessive ECM deposition during hepatic carcinogenesis, which was blocked by TM4SF5 inhibition, using an anti-TM4SF5 small compound. TM4SF5 inhibition or suppression downregulated co-stimulatory activation ligands in hepatocytes, which caused decreased cognate receptor expression on NK cells and eventually led to immune surveillance of TM4SF5-positive tumors. Thus, TM4SF5 is a potential target for NK cell-related immunotherapy against HCC.

## Materials and methods

### Cells

Human hepatocarcinoma cell lines that lacked TM4SF5 expression (SNU449 and empty-vector control SNU449C_p_ cells) or that expressed wild-type (WT) TM4SF5 [(exogenous expression) SNU449T_7_, (endogenous expression) HepG2, Huh7, and HepG2 cells] were used for in vitro experiments and were previously described [17]. Cells were purchased from either the Korean Cell Bank (Seoul, Korea) or American Type Culture Collection (ATCC, Manassas, VA, USA). Hepatocytes were cultured in Roswell Park Memorial Institute (RPMI) 1640 Medium or Dulbecco’s Modified Eagle Medium (DMEM) (Cytiva, Marlborough, MA, USA) supplemented with 10% fetal bovine serum (FBS) and 1% penicillin/streptomycin (GenDEPOT Inc., Barker, TX, USA) at 37 °C in 5% CO_2_. Cells were stably infected with empty pLJM1-EGFP (Addgene) or TM4SF5-encoding pLJM1-EGFP lentiviral vectors. Human NK92 cells were cultured in α-Minimum Essential Medium (MEM) (Invitrogen, Grand Island, NY, USA) containing 200 U/mL recombinant human interleukin (IL)-2 (PeproTech, Rocky Hill, NJ, USA), 12.5% FBS, and 12.5% fetal horse serum (GenDEPOT Inc.). Stable cells were cultured in RPMI 1640 (WelGene) containing 10% FBS, geneticin (G418; 250 μg/mL), and antibiotics (Invitrogen, Grand Island, NY, USA). Cells were passaged every 3–4 days at ratios specified by the suppliers. Cells were monitored for mycoplasma contamination.

### Transfection and infection

The shRNA or cDNA expression plasmids were transfected for 48 h using Lipofectamine RNAiMAX or Lipofectamine 3000 (Thermo Fisher Scientific, Waltham, MA, USA), respectively, according to the manufacturer’s protocols. Alternatively, cells were infected with lentivirus for shRNA or cDNA expression for 24 h. Lentiviruses encoding shRNA against TM4SF5 (5′-CCGGACCATGTGTACGGGAAAATGTGCCTCGAGGCACATTTTCCCGTACACATGGTTTTTTG-3′ for sequence #2, 5′-CCGGCCATCTCAGCTTGCAAGTCCTCGAGGACTTGCAAGCTGAGATGGTTTTTG-3′ for sequence #4) were prepared using lentiviral vector pLKO.1 (Addgene). Infected cells were selected with puromycin (2 μg/mL, GenDEPOT).

### Mice

Age-matched WT and *Tm4sf5*-knockout (*Tm4sf5*^−/−^) C57BL/6 male mice as well as WT and *TM4SF5*-transgenic (Tg^TM4SF5^) FVB/N male mice were used for in vivo experiments. Tg^TM4SF5^ FVB/N mice with a CMV promoter on the Tg construct for expression throughout the entire mouse were generated by breeding WT FVB/N and Tg^TM4SF5^ C57BL/6 mice [22] for 10 generations. The FVB/N strain was selected because it is susceptible to spontaneous development of lung cancer, rather than cancer of any other organs, including the liver. Furthermore, the ratio of liver tumors is lower in FVB/N mice than in other strains [23]. *Tm4sf5*^−/−^ C57BL/6 mice were generated via embryo injection and transfer to normal, healthy female C57BL/6 recipient mice (Macrogen, Seoul, Korea), as described previously [24]. Briefly, the exon 1 of C57BL/6N-Tac *Tm4sf5* gene by Cas9 proteins single guide RNAs (50 μg, 5′-GAGGTTGCCGTCCGTCCAGGTGG-3′ and 5′-GCTGAGGTTGCCGTCCGTCCAGG**-**3′ was targeted, and its deletion was proved by genotyping using primers for mouse-*Tm4sf5* (forward 5′-CCAAGCCTCCCACCTGTTA-3′, reverse 5′-GCTCCAGCATTCTCACCATC-3′ for KO_exon1_). We further regularly backcrossed the KO mice with WT mice. F1 heterozygous littermates (*Tm4sf5*^*−/*+^) were bred to generate homozygous mice (*Tm4sf5*^*−/−*^). The littermates of WT, *Tm4sf5*^−/−^ C57BL/6, WT or Tg^TM4SF5^ FVB/N mice (isolated from a variety of different C57BL/6 or FVB/N litters, respectively) were used in experiments after randomized assignment following genotyping.

### Chemically induced animal models

Four-week-old mice (BALB/c) were purchased from Orient. Co. Ltd (Seungnam, Korea). The mice were housed in a specific pathogen-free room with controlled temperature and humidity. Five-week-old mice (n ≥ 5) were injected intraperitoneally with or without CCl_4_ (Sigma-Aldrich, St. Louis, MO, USA; 1 mg/kg body weight, 1 time in the beginning of the experiment), or diethylnitrosamine **(**DEN, Sigma-Aldrich; 50 mg/kg body weight, one time/week**)** in 40% olive oil for 16 or 27 weeks, respectively. For 4′-(*p*-toluenesulfonylamido)-4-hydroxychalcone (TSAHC) [25] administration [50 mg/kg in 40% dimethylsulfoxide (DMSO)], mice were injected intraperitoneally for 16 weeks (every 2 or 3 days per week). TSAHC injection was started either at week 0 (DEN + TSAHC1 group) or week 10 (DEN + TSAHC2 group) for overall 27 week-treatment for DEN-treated liver cancer model. Alternatively, age-matched (2-week-old) WT and *Tm4sf5*^−/−^ mice (*n* ≥ 5) were injected intraperitoneally with or without DEN (25 mg/kg body weight) at day 0. TSAHC was also injected intraperitoneally twice a week (5 mg/kg body weight). After 45 weeks, the mice were sacrificed for analyses. The mice were euthanized with ether, and the liver and spleen tissues were resected. One piece of liver tissue was immediately frozen in liquid N_2_, whereas a second piece was embedded in paraffin or alternatively used for primary hepatocyte preparation. Plasma samples were also collected for plasma parameter analyses.

### Western blot analysis

Sub-confluent cells in normal culture media were transfected or infected with control or specific siRNA, shRNA vectors, or virus encoding the indicated molecules for 48 or 24 h, respectively, in the presence of vehicle or TSAHC [26] treatment. Animal liver tissues were harvested for whole-cell or tissue extraction using a modified RIPA buffer, as previously described [17, 21]. The primary antibodies (generally at 1:1000 dilution) used included antibodies against laminins, pY^397^FAK, MICA/B (Abcam, Cambridge, UK), p-ERKs, ERKs (Cell Signaling Technology. Danvers, MA, USA), pY^705^STAT3 (Millipore, Billerica, MA, USA), β-actin, α-tubulin, SLAMF7 (CS1), MICA/B, STAT3, pY^577^FAK (Santa Cruz Biotechnology, Santa Cruz, CA, USA), FAK (BD Transduction Laboratories, Bedford, MA, USA), collagen I (Acris Antibodies, Herford, Germany), and TM4SF5 [17]. The TM4SF5_C-ter_ (epitope region of RKKQDTPH^197^) antibody was custom designed (Pro-Sci, Poway, CA, USA).

### Natural killer cell cytotoxicity assay

Evaluation of NK cell cytotoxic activity was performed using a lactate dehydrogenase (LDH) cytotoxicity assay kit (Cytotoxicity Detection Kit Plus, Roche). Human HCC cell lines (Huh7 or HepG2 cells with endogenous TM4SF5 expression; target cancer cells) were seeded (0.5 × 10^4^ cells/well; 50 μL) into a 96-well plate in triplicate with assay medium (RPMI 1640 with 1% FBS). Human NK92 cells pre-treated with IL-2 (effector cells) were adjusted to a concentration of 1 × 10^6^ cells/mL in assay medium, and then two-fold dilutions of the NK cells were prepared (1 × 10^6^, 0.5 × 10^6^, 0.25 × 10^6^, and 0.125 × 10^6^ cells/mL). NK cell suspension was pipetted into the wells of the 96-well plate in triplicate at effector (E) to target (T) cell ratios (E:T) of 1.25:1, 2.5:1, 5:1, and 10:1 to see the TM4SF5-dependent effects. Cells were incubated in the 96-well plate for 4 h at 37 °C in 5% CO_2_. LDH released from the dead target cells was measured using an ELISA reader at 492–690 nm. Percentage (%) of cytotoxicity was calculated as follows: [LDH (effector-target cell mix) − LDH (effector cell) − LDH (target cell low control)]/[LDH (target cell high control) − LDH (target cell low control)] × 100. After co-culturing, non-adherent NK cells were collected and centrifuged at 150×*g* for 5 min to remove the target cancer cell debris. Quantitative RT-PCR was then performed to analyze the ligand or receptor mRNA levels of the hepatocytes or NK92 cells, respectively.

### Murine splenic and intrahepatic immune cell analysis

Mouse spleens and livers were dissected, and cell suspensions were generated by mechanical disruption through 70- or 100-μm nylon mesh filters, respectively. Immune cells were isolated from the spleens and suspended in RPMI 1640 (with 2% FBS). The immune cell suspension was then centrifuged at 480×*g* for 8 min, and the supernatant was removed. The cell pellets were resuspended in ammonium-chloride-potassium (ACK) buffer (RBC lysis buffer, Thermo Fisher Scientific, Cat. No: A1049201) and incubated for 5 min at 37 °C. Next, the cell suspension was centrifuged at 480×*g* for 8 min, and the supernatant was removed. The cell pellets were washed twice using FACS staining buffer (BD Bioscience, Cat. No: 554657) and centrifuged at 480×*g* for 8 min. The cell pellet suspensions with the staining buffer were then incubated with Fc Block (BD Bioscience, Cat. No: 564219) at 4 °C for 15 min to block the lymphocyte FcγII/III receptors. Intrahepatic immune cells were enriched using a 37.5% Percoll (GE Healthcare) gradient. Erythrocytes were lysed with red blood cell (RBC) lysis buffer (Invitrogen). To assess IFN-γ, perforin 1 (PRF1), and granzyme B (GZMB) expression, lymphocytes were incubated with 5 ng/mL phorbol 12-myristate 13-acetate (PMA, Sigma), 500 ng/mL ionomycin (Sigma), and 10 ng/mL monensin (Sigma), respectively, for 4 h at 37 °C in 5% CO_2_. Lymphocyte FcgII/III receptors were blocked with Fc Block (BD Bioscience), and surface antigens were stained with conjugated and biotinylated monoclonal antibodies (mAbs). Splenic or intrahepatic immune cells were then fixed and permeabilized using Cytofix/Cytoperm (BD Bioscience), and intracellular antigens were detected using conjugated and biotinylated mAbs at 4 °C for 30 min. Phycoerythrin (PE)-conjugated anti-human PRF1 allophycocyanin (APC)-conjugated anti-mouse GZMB, PE-conjugated anti-mouse PRF1 fluorescein isothiocyanate (FITC)-conjugated anti-mouse CD45, PE-conjugated anti-mouse CD4, APC/Cy7-conjugated anti-mouse CD3, FITC-conjugated anti-mouse CD8a (BioLegends), BV421-conjugated anti-mouse CD8a, and BV421-conjugated anti-mouse NK1.1 antibodies (BD Bioscience) were used for staining. Flow cytometry analysis was performed on a FACS LSR Fortessa X-20 (BD) and analyzed with FlowJo version 10.6.1. Graphic data were presented the mean ± standard error of the mean (SEM).

### Immunohistochemistry and tissue staining

Liver tissues from human liver cancer patients, Tg^TM4SF5^ FVB/N mice, or C57BL/6 (WT or *Tm4sf5*^−/−^ KO) mice treated with or without DEN and in the absence or presence of TSAHC were processed for immunohistochemistry. Liver sections were fixed with 3.7% formaldehyde and embedded in paraffin. The fixed liver sections were deparaffinized and rehydrated. Antigen retrieval was performed with heat-induced epitope retrieval (HIER) using sodium citrate buffer (pH 6.0). Quenching and blocking were performed using 3% H_2_O_2_ in distilled water and 1% normal goat serum in phosphate-buffered saline (PBS). Antigens were stained using the avidin–biotin complex (ABC) method (VECTASTAIN Elite ABC HRP Kit, Vector) and were detected using 3,3′-diaminobenzidine (DAB) stain (Vector). Antibodies against TM4SF5 [17], collagen I (Acris Antibodies), pY^705^STAT3 (Cell Signaling Technology), normal rabbit or mouse IgG, α-SMA (Sigma-Aldrich), α-fetoprotein (AFP), CD34, Ki67, laminins (Abcam), α-l-fucosidase [FUCA (AFU)], and laminin γ2 (Santa Cruz Biotechnology) were used for immunostaining. Ten random images per slide were saved using a digital slide scanner (MoticEasyScan, Motic, British Columbia, Canada). The tissues were also processed with Masson’s trichrome as well as hematoxylin and eosin stains, as previously described [27].

### Polymerase chain reaction

Total RNA from animal liver tissues or cells were isolated using TRIzol reagent (Invitrogen), and total RNA isolated from the samples was treated with ReverTra Ace qPCR RT Master Mix (TOYOBO) with gDNA Remover to generate complementary DNA (cDNA). The cDNA was subjected to RT-PCR using the Dream Taq Green PCR Master Mix (Thermo Scientific, San Jose, CA, USA). Quantitative RT-PCR was prepared with LaboPass EvaGreen Q Master (Cosmo Genetech, Seoul, Korea) and performed with the CFX Connect Real-Time PCR Detection System (Bio-Rad, Hercules, CA, USA). The mRNA levels were normalized against 18S ribosomal RNA, using the ddCq method. The CFX Maestro Software (Sunnyvale, CA, USA) was used to analyze the data. Primers were purchased from Cosmo Genetech (Seoul, Korea). The primer sequences are shown in Table 1.

**Table 1:** The primer sequences for RT-PCR or q-PCR in the current study

Gene name	Sequence	
	Forward (5′ → 3′)	Reverse (5′ → 3′)
Human		
*TM4SF5*	CTTGCTCAACCGCACTCTAT	ATCCCACACAGTACTATCTCCA
*hSLAMF6*	GTCCAGAAATCCACGTGACTAA	GTAAGAGCCTGTGTCTTCCATC
*hSLAMF7*	TGGGTCTGCAGAGCAATAAG	CAGGGCCTTCCAGGTATAAAT
*hMICA*	CCTTGGCCATGAACGTCAGG	CCTCTGAGGCCTCGCTGCG
*hMICB*	ACCTTGGCTATGAACGTCACA	CCCTCTGAGACCTCGCTGCA
*hULBP1*	TTTCCTTAAAGGGCAACTGCT	AGGAACTGCCAAGATCCTCT
*hULBP2*	CATTACTTCTCAATGGGAGACTGT	TGTGCCTGAGGACATGGCGA
*hULBP3*	CTGATGCACAGGAAGAAGAG	TATGGCTTTGGGTTGAGCTAA
*hULBP5*	GACTCGCGTGACTTTACCTATC	GGCTGCGCCGTTATTTATTG
*hCD111*(*Nectin-1*)	ATCCTGCTGGTGTTGATTGT	TGCTTCTTGGTGCTGTAGTC
*hCD112*(*Nectin-2*)	TTCCCTGGATCTAGAGGATGAG	CTTTGCCCTGGTAGGAATCA
*hCD113*(*Nectin-3*)	TTGCTAGAGGAAGGCGAATTAC	ACCGAAACTTCAGGAGCATAC
*hCD155*(*PVR*)	AGGTATCCATCTCTGGCTATGA	CCATGGTCGTGCTCCAATTA
*hCADM1*	GCTTCTGCTGTTGCTCTTCT	CTCGATCACTGTCACGTCTTTC
*hKlrk1*(*NKG2D*)	CAGCAAAGAGGACCAGGATTTA	GTTAGTAGGTTGGGTGAGAGAATG
*hPRF1*	CCCAGTGGACACACAAAGGTT	TCGTTGCGGATGCTACGAG
*hGZMB*	CTTCCTGATACGAGACGACTTC	CGGCTCCTGTTCTTTGATATTG
*hCD107a*	CGTCAGCAGCAATGTTTATGG	CATGTTCTTAGGGCCACTCTT
*hTNF-α*	GAGTGACAAGCCTGTAGCCCATGTTGTAGCA	GCA ATG ATCCCAAAGTAGACCTGCCCAGACT
*hFasL*	CATTTAACAGGCAAGTCCAACTC	CACAAGGCCACCCTTCTTAT
*hTNFSF10*(*TRAIL*)	CAGAGAGTAGCAGCTCACATAAC	CCTTGATGATTCCCAGGAGTTT
Mouse		
*Tm4sf5*	GTCTTCTCCTCCGCCTTTG	GGTAGTCCCACTTGTTGTCTATT
*F4/80*	ACCACAATACCTACATGCACC	AAGCAGGCGAGGAAAAGATAG
*Mcp-1*	TTAAAAACCTGGATCGGAACCAA	GCATTAGCTTCAGATTTACGGGT
*Tnf-α*	CCCTCACACTCAGATCATCTTCT	GCTACGACGTGGGCTACAG
*Il-6*	GAGGATACCACTCCCAACAGACC	AAGTGCATCATCGTTGTTCATACA
*Cd34*	CAGGAGAAAGGCTGGGTGAA	GTTGTCTTGCTGAATGGCCG
*Ki67*	AAAGGCGAAGTGGAGCTTCT	TTTCGCAACTTTCGTTTGTG
*Gapdh*	GACATGCCGCCTGGAGAAAC	AGCCCAGGATGCCCTTTAGT

### Analysis of public RNA expression data

The mRNA expression levels of some genes were searched in the public Gene Expression Omnibus (GEO, https://www.ncbi.nlm.nih.gov/geo/) database (accession numbers GSE6764, GSE14520, and GSE76427). The mRNA expression levels were extracted using the built-in function, ‘Analyze with GEO2R’. The specific identification numbers for *TM4SF5* and *SOCS1* were 8003939 and 7999423 from GSE6764, respectively, and *TM4SF5* and *LAMC2* were ILMN 2167808 and ILMN 1653824 from GSE76427, respectively. Public identification numbers of GSE14520 were for *TM4SF5* (ID No. 4507538), *SLAMF7* (12711663), *MICA* (4557750), *ULBP1* (13376825), and *ULBP2* (13376823). The available mRNA expression levels for the indicated groups were further analyzed for comparison. The Mann–Whitney *U* test was applied for statistical analysis using GraphPad Prism 7.0. A box-and-whisker plot was drawn using GraphPad Prism 7.0.

### Study approval

Human liver tissues were collected after obtaining informed consent from the liver cancer patients at the Seoul National University under institutional review board (IRB)-approved protocols (Seoul, Korea). All animal procedures were performed in accordance with the Seoul National University Laboratory Animal Maintenance Manual and were approved by the IRB of the Institute of Laboratory Animal Resources Seoul National University (SNU-IACUC) (SNU-140423-11-7, SNU-130911-2-3, SNU-170920-9, SNU-190122-6-3).

### Statistics

Statistical analyses were performed using Prism Software (GraphPad 7.0, La Jolla, CA, USA). Two-way analysis of variance (ANOVA), ordinary one-way ANOVA, an unpaired, one-tailed Mann–Whitney *U* test, or unpaired, two-tailed Student’s *t* tests were performed to determine statistical significance. A *p* value < 0.05 was considered statistically significant.

## Results

### TM4SF5 mediated hepatic carcinogenesis

**Fig. 1:**
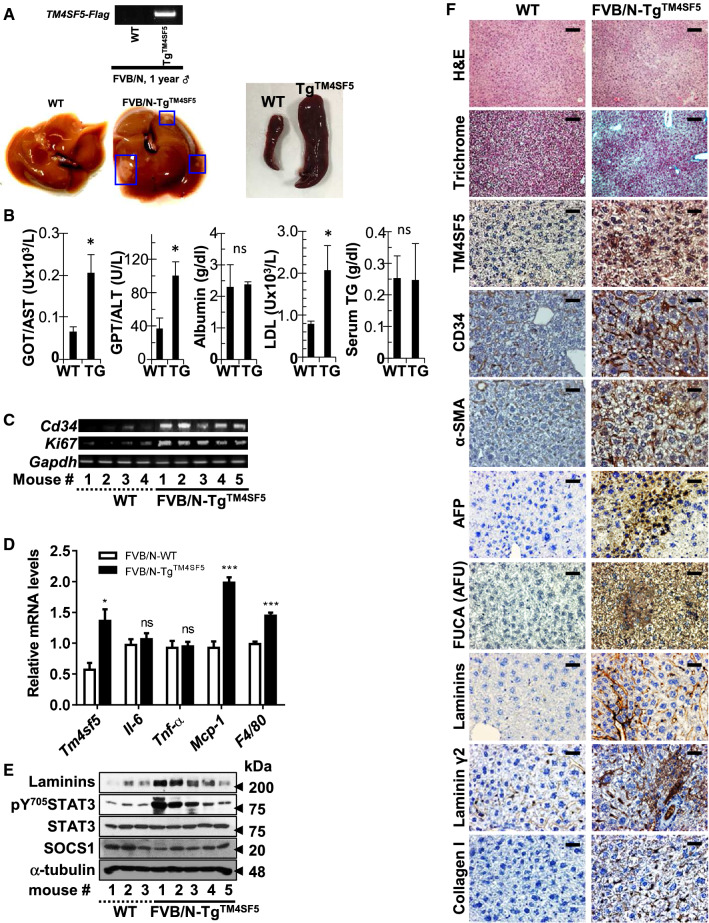
TM4SF5-mediated hepatic carcinogenesis in TM4SF5-transgenic mice. **A** Liver nodules in 1-year-old FVB/N-Tg^TM4SF5^ mice (*n* = 2/7) compared with age-matched wild-type (WT) mice (*n* = 0/7). Tg^TM4SF5^ mice showed enlarged spleens compared with age-matched WT mice. **B** The livers of 1-year-old FVB/N-Tg^TM4SF5^ mice were examined for aspartate aminotransferase (AST), alanine aminotransferase (ALT), low-density lipoprotein (LDL), albumin, and serum triglyceride (TG) levels and were compared with those of age-matched WT mice. **C**, **D** The mRNA levels of the livers of 1-year-old FVB/N-Tg^TM4SF5^ and age-matched WT mice were analyzed for the indicated molecules. For statistical significance, * and *** represent *p* ≤ 0.05 and *p* ≤ 0.001, respectively, and ns indicates no significance, following Student’s *t* tests. **E**, **F** The livers of 1-year-old FVB/N-Tg^TM4SF5^ and age-matched WT mice were processed for immunoblot (**E**) and immunohistochemical (**F**) analyses. The livers of Tg^TM4SF5^ mice showed enhanced expression of hepatocellular carcinoma markers, including CD34, α-fetoprotein (AFP), and α-l-fucosidase [FUCA (AFU)], in addition to pY^705^STAT3 and extracellular matrix factors, including collagen I, laminin γ2, and laminins. Data shown represent three isolated experiments

**Fig. 2:**
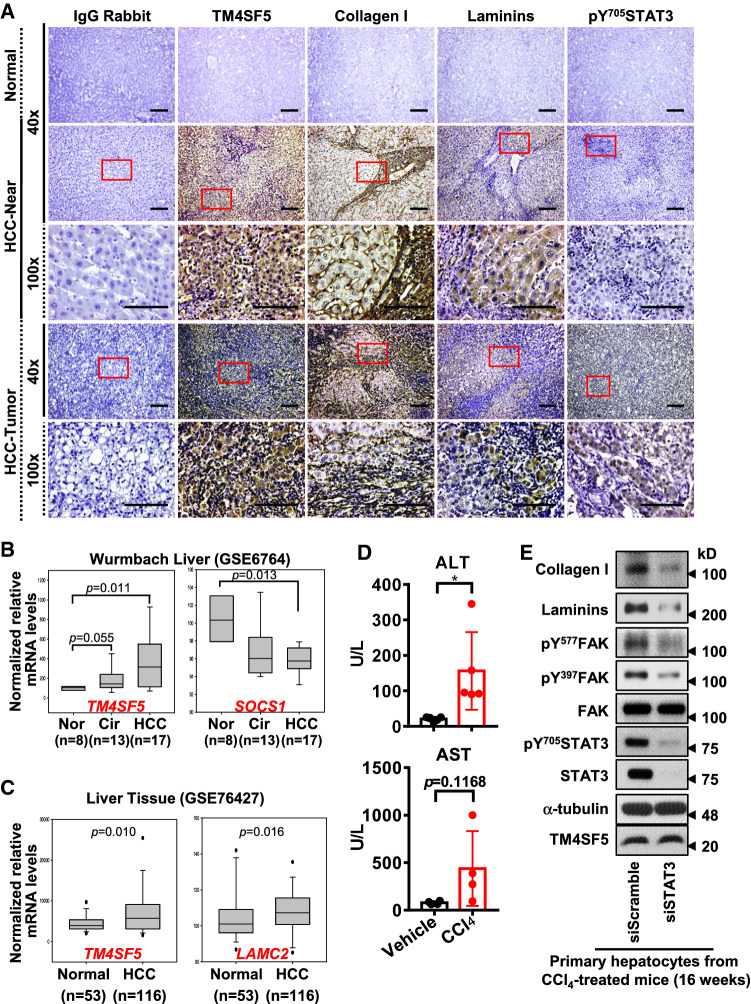
Clinical relevance of TM4SF5-mediated STAT3 activity, extracellular matrix production, and hepatic cancer. **A** Human liver cancer tissues were processed for immunohistochemical analysis and showed higher levels of TM4SF5, pY^705^STAT3, collagen I, and laminins, compared with normal surrounding liver tissues. **B**, **C** Gene expression profiles of liver cirrhosis [normal (Nor) vs. cirrhosis (Cir) vs. HCC] from GSE6764 were analyzed and showed *TM4SF5*-positive cirrhosis (*p* = 0.055) and HCC (*p* = 0.011) and *SOCS1*-reduced cirrhosis and HCC (*p* = 0.013) groups. *p-*values were calculated by the Dunnett’s test and ordinary one-way ANOVA. Other analyses of hepatocellular carcinoma (HCC) (normal vs. HCC) from GSE76427 showed *TM4SF5*-positive (*p* = 0.010) and *LAMC2*-promoted HCC (*p* = 0.016) groups compared with normal groups. *p-*values were calculated by the Mann–Whitney *U* test. **D** Primary hepatocytes were prepared from mice treated with CCl_4_ for 16 weeks as an animal cirrhosis model and were transfected with STAT3-specific or scrambled control siRNA for 48 h before harvesting whole-cell extracts for immunoblot analysis. Data shown represent three independent experiments

Because C57BL/6-Tg^TM4SF5^ mice exhibited nonalcoholic steatohepatitis (NASH)-associated fibrotic livers [24], we determined whether TM4SF5 overexpression in a disease-susceptible mouse strain (i.e., FVB/N [23]) caused an enhanced malignant cancer phenotype. The livers of 1-year-old FVB/N-Tg^TM4SF5^ mice were analyzed to test this hypothesis. Out of seven mice, we found two with hepatic nodules, suggesting hepatic carcinogenesis. Interestingly, the Tg^TM4SF5^ mice also exhibited enlarged spleens compared with age-matched wild-type (WT) mice (Fig. 1A). The FVB/N-Tg^TM4SF5^ mice showed higher aspartate aminotransferase (AST), alanine aminotransferase (ALT), and low-density lipoprotein (LDL) levels with comparable levels of albumin and serum triglycerides (TGs) compared with age-matched WT mice (Fig. 1B). Furthermore, *CD34* and *Ki67* mRNA levels were higher in the livers of FVB/N Tg^TM4SF5^ mice than in age-matched WT mice (Fig. 1C), indicating cell proliferation. Interestingly, liver monocyte chemoattractant protein 1 [*Mcp-1* (chemokine ligand 2, *Ccl2*)] and *F4/80* mRNA levels were also elevated in the Tg^TM4SF5^ mice compared with age-matched WT mice, indicating immune system involvement (Fig. 1D). Indeed, serum MCP-1 was reported to be positively correlated with α-fetoprotein (AFP) levels, suggesting it as another tumor marker in HCC [28]. The levels of laminins and pY^705^STAT3 (Fig. 1E), collagen I, α-smooth muscle actin (SMA), laminins, laminin γ2, and TM4SF5 expression levels were elevated in FVB/N-Tg^TM4SF5^ mice compared with age-matched WT mice, in addition to CD34, AFP, α-l-fucosidase [FUCA (AFU)], which are HCC markers [29–31], hepatic damage, and collagen I deposition (Fig. 1F). These observations support the hypothesis that the livers of FVB/N Tg^TM4SF5^ mice become cancerous. Moreover, immunostaining of liver tissues from HCC patients showed substantially elevated levels of TM4SF5, laminins, collagen I, and pY^705^STAT3 in peritumoral (presumably with NASH and fibrosis phenotypes) and tumor regions compared with normal regions (Fig. 2A). Importantly, laminin-positive cells were predominantly appeared to be hepatocytes with large cytosolic spaces, which were different from collagen I-positive cells (Fig. 2A), suggesting a role of TM4SF5-induced laminins in hepatocytes during hepatic carcinogenesis. In addition, public Gene Expression Omnibus (GEO) data analysis showed that *TM4SF5* expression was highly elevated in HCC patients, and these elevated levels were negatively correlated with *SOCS1* expression in both cirrhosis and HCC patients compared with the normal population (Fig. 2B). Further, increased *TM4SF5* expression was also positively correlated with enhanced *LAMC2* expression (Fig. 2C). Indeed, STAT3 suppression in the primary hepatocytes of CCl_4_-induced cirrhotic livers (16-week treatment) decreased collagen I and laminin expression in addition to STAT3 and FAK phosphorylation, as downstream effectors of TM4SF5 (Fig. 2D). These findings suggest that TM4SF5/STAT3-mediated expression of ECM factors may be involved in fibrosis/cirrhosis and subsequent hepatic carcinogenesis.

### TM4SF5 inhibitor abolished diethylnitrosamine-induced hepatic carcinogenesis

**Fig. 3:**
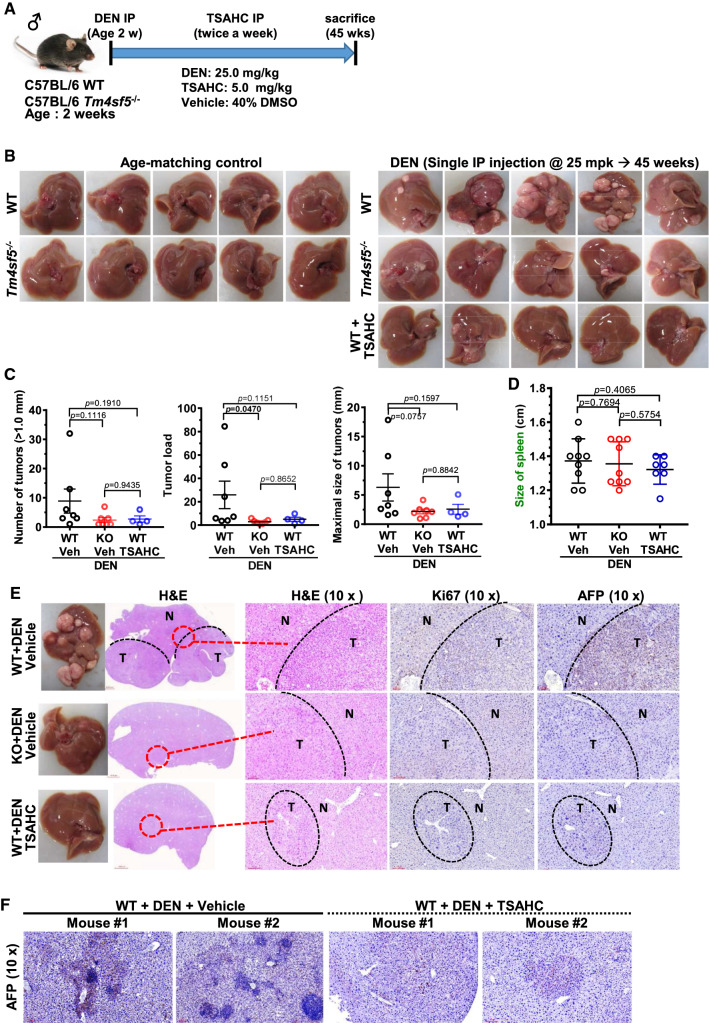
TM4SF5 expression and activity-dependent granulomatous inflammation during diethylnitrosamine-induced hepatic carcinogenesis. **A** Age-matched WT or *Tm4sf5*^−/−^ male C57BL/6 mice (2 weeks old) were intraperitoneally injected once with diethylnitrosamine (DEN, 25 mg/kg body weight) and twice per week with 4′-(*p*-toluenesulfonylamido)-4-hydroxychalcone (TSAHC, 5.0 mg/kg body weight) for 45 weeks. After 45 weeks on a normal diet ad libitum, the mice were sacrificed for analysis. Age-matched WT and knockout mice (*n* = 6), DEN-treated WT and knockout mice (*n* = 7), and DEN/TSAHC-treated WT mice (*n* = 5) were examined in parallel. **B** Five representative liver images were shown for each group. **C** Tumors with a diameter larger than 1.0 mm were counted, and the largest tumor in each animal was determined. Age-matched WT and knockout animals without DEN treatment did not form any tumors. One outlier (second, left) from the DEN/TSAHC-treated WT group was not included in the graphs. *p*-values were calculated by Sidak’s test and one-way ANOVA. A *p*-value ≤ 0.05 was considered statistically significant. **D** After 45 weeks, spleens were also collected from each animal and weighed. Data are represented as the mean ± standard deviation. *p*-values were calculated by Sidak’s test and one-way ANOVA. A *p*-value ≤ 0.05 was considered statistically significant. **E**, **F** Liver tissues were processed for hematoxylin and eosin staining or immunohistochemical analysis for Ki67 and AFP protein expression levels. N and T depict normal and tumor regions, respectively (**E**). More significant granuloma-like lesions were observed in the tumor tissues of DEN-treated WT mice compared with DEN/TSAHC-treated WT mice (**F**). Data shown represent three independent experiments

Next, we investigated the roles of the immune system in TM4SF5-mediated hepatic carcinogenesis using *Tm4sf5*^−/−^ and age-matched WT mice treated with or without diethylnitrosamine (DEN) in the absence or presence of TM4SF5 inhibitor 4′-(*p*-toluenesulfonylamido)-4-hydroxychalcone (TSAHC) treatment. The mice were treated once with DEN and twice a week with TSAHC. After 45 weeks, the livers of the mice were analyzed, as depicted in the scheme (Fig. 3A). The livers of the mice exhibited dramatic differences in nodule formation depending on TM4SF5 expression and function (i.e., activity). Age-matched WT and knockout mice without DEN treatment did not form liver nodules. However, WT mice treated with DEN showed dramatic nodule formations, which was abolished by additional TSAHC treatment (Fig. 3B). Indeed, *Tm4sf5*^−/−^ mice did not show substantial nodule formation, with being similar in the DEN-treated WT mice with TSAHC treatment (Fig. 3B). Quantitative analysis in terms of the number of tumors larger than 0.1 mm in diameter, tumor load (as the sum of tumor diameter of all tumors with diameters > 1.0 mm), and the maximal tumor size resulted in greater values for DEN-treated WT mice than DEN-treated knockout mice and DEN/TSAHC-treated WT mice, although the change tendencies were statistically significant (*p* = 0.0470) or insignificant (*p* = 0.0757–0.1910) (Fig. 3C). Unlike the FVB/N-Tg^TM4SF5^ mice, DEN-treated WT mice did not have significantly larger spleen sizes compared with DEN-treated knockout mice and DEN/TSAHC-treated WT mice (*p* = 0.4065, Fig. 3D).

Hematoxylin and eosin (H&E) staining and immunohistochemical analyses of the liver tissues showed dramatically different tumor lesions among the mice. More aggressive tumor lesions were shown in the DEN-treated WT mice by positive Ki67 and AFP staining, whereas DEN-treated knockout and DEN/TSAHC-treated WT mice showed fewer tumors with less positive or negative Ki67 and AFP staining (Fig. 3E). Interestingly, we found significant formation of noninfectious granulomatous inflammation (with immune cell infiltrates) near the portal tracks with positive AFP staining in the DEN-treated WT mice, which was abolished by additional TSAHC treatment (Fig. 3F). These observations indicate that TM4SF5 activation may be involved in the inflammation during DEN-induced hepatic carcinogenesis and that inhibition of TM4SF5 by TSAHC may interfere with the DEN-mediated effects.

### Natural killer cells were inhibited by TM4SF5 during diethylnitrosamine-induced hepatic carcinogenesis

**Fig. 4:**
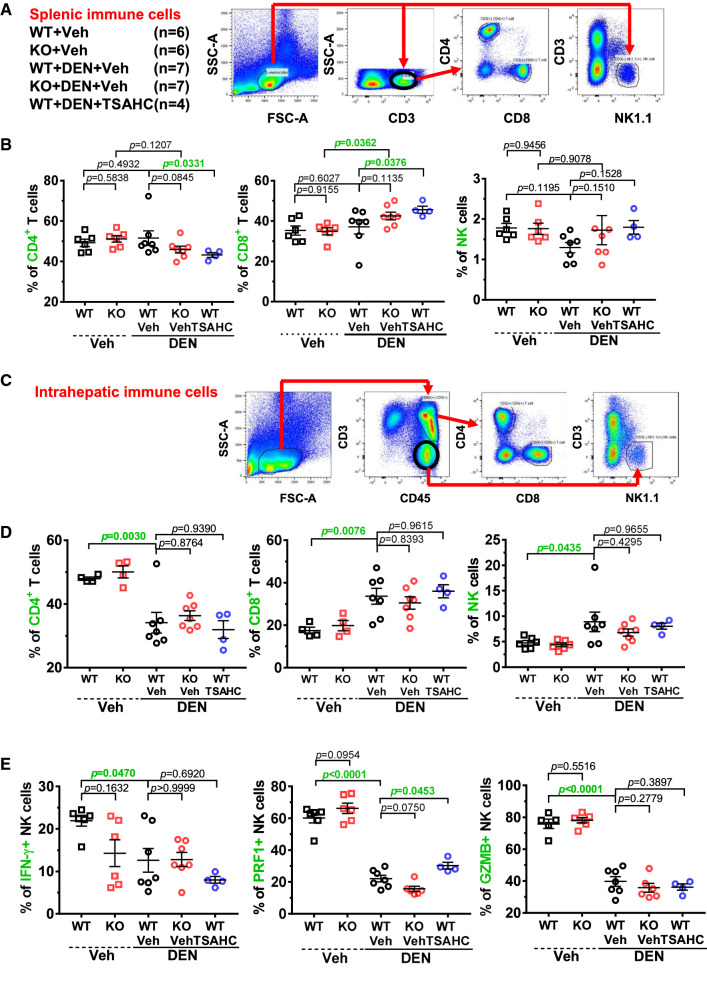
Natural killer cell involvement in diethylnitrosamine-induced, TM4SF5-dependent hepatic carcinogenesis. Spleen and liver tissues from WT and *Tm4sf5*^−/−^ animals with or without diethylnitrosamine (DEN) treatment and in the absence or presence of 4′-(*p*-toluenesulfonylamido)-4-hydroxychalcone (TSAHC) co-treatment (described in Fig. 3A) were processed for splenic and hepatic immune cell population analysis by flow cytometry. **A**, **B** One outlier was excluded from the DEN/TSAHC-treated WT group. Splenic tissues for each experimental group were processed for immune cell population analysis using CD3^+^/CD4^+^ [T-helper (Th) cells], CD3^+^/CD8^+^ (T cells), and CD^3−^/NK1.1^+^ [natural killer (NK) cells] cell markers (**A**). The cell populations were graphed, and *p* values were calculated by Sidak’s test and one-way ANOVA. A *p*-value ≤ 0.05 was considered statistically significant (**B**). **C**, **D** One outlier was excluded from the DEN/TSAHC-treated WT group. Hepatic tissues for each experimental group were processed for immune cell population analysis using CD3^+^/CD4^+^, CD3^+^/CD8^+^, and CD3^−^/CD45^+^/NK1.1^+^ cell markers (**C**). The intrahepatic cell populations were graphed, and *p* values were calculated by Dunnett’s test and one-way ANOVA. A *p*-value ≤ 0.05 was considered statistically significant (**D**). **E** NK cells were analyzed for cytokine or granule secretion via antibody staining and flow cytometry. *p* values were calculated by Sidak’s test and one-way ANOVA. A *p*-value ≤ 0.05 was considered statistically significant. Data represent three independent experiments or measurements

Because the hepatic granuloma-like lesions within the DEN-induced, AFP-positive tumors exhibited immune cell infiltration, we investigated the potential mechanism of the immune cells in carcinogenesis. First, we used immunostaining and FACS to analyze the population of splenic immune cells for CD3^+^/CD4^+^ (T-helper cells, Th), CD3^+^/CD8^+^ (cytotoxic T cells), and CD3^−^/NK1.1^+^ (NK cells) cell markers (Fig. 4A). Splenic CD4^+^ Th-cell populations were not significantly different between vehicle- and DEN-treated WT or KO mice; however, DEN/TSAHC-treated WT mice showed significantly (*p* = 0.0331) lower CD4^+^ Th-cell populations compared with DEN-treated WT mice (Fig. 4B, left). CD8^+^ T-cell populations were not differential between vehicle-treated WT and KO mice (*p* = 0.9155), and significantly increased in KO mice upon DEN treatment (*p* = 0.0362) or in DEN-treated WT mice upon further TSAHC treatment (*p* = 0.0376) (Fig. 4B, middle). Therefore, CD4^+^ Th- and CD8^+^ T-cell populations appeared not to be significantly involved in the immune effects during TM4SF5 expression (e.g., WT vs. KO) but in TM4SF5 function/activity-dependent carcinogenesis (e.g., WT-DEN vs. WT-DEN/TSAHC). However, consistent with the tumor load patterns, DEN-treated WT mice showed a slight but insignificant reduction in the population of NK cells compared with vehicle-treated WT mice (*p* = 0.1195), and DEN-treated KO mice and DEN/TSAHC-treated WT mice exhibited slight but statistically insignificant recoveries in the splenic NK cell population (*p* = 0.1510 or 0.1528, respectively), suggesting a possible involvement (though insignificant presumably due to smaller sample numbers) of NK cells in response to TM4SF5 blockade with TSAHC during DEN-induced carcinogenesis (Fig. 4B, right).

We also examined the intrahepatic immune cell populations via immunostaining and FACS for CD3^+^/CD4^+^ Th-cells, CD3^+^/CD8^+^ T-cells, and CD3^−^/CD45^+^/NK1.1^+^ NK cells (Fig. 4C). Unlike splenic CD4^+^ Th-cells, hepatic CD4^+^ Th-cells were reduced in DEN-treated WT mice and were not recovered by TSAHC treatment or *Tm4sf5*-knockout (Fig. 4D, left). Furthermore, hepatic CD8^+^ T-cell populations were increased in WT mice upon DEN treatment (*p* = 0.0076), which were not changed by TSAHC treatment or *Tm4sf5*-deficiency (Fig. 4D, middle). Additionally, DEN-treated WT mice showed higher populations of NK cells than vehicle-treated WT mice (*p* = 0.0435), which was similar to the levels of DEN-treated KO mice (*p* = 0.4295) or DEN/TSAHC-treated WT mice (*p* = 0.9655, Fig. 4D, right). Therefore, intrahepatic immune cells, including Th, cytotoxic T, and NK cell populations, did not appear to be correlated with the TM4SF5-dependent carcinogenesis in DEN-treated mice. However, splenic NK cell numbers might still have roles in DEN-induced carcinogenesis, depending on TM4SF5 activity, although their effects were not statistically insignificant presumably due to small sample numbers (Fig. 4B).

We therefore analyzed further intrahepatic NK cell activity by immunostaining for perforin, granzyme, and cytokine IFN-γ. Although DEN-treated WT mice exhibited lower levels of IFN-γ and granzyme than vehicle-treated WT mice, their levels were not significantly different by additional TSAHC treatment (*p* = 0.6920 and *p* = 0.3897, respectively) and from those in DEN-treated KO mice (*p* > 0.9999 and *p* = 0.2779, respectively) (Fig. 4E). Meanwhile, only the perforin levels in WT mice were significantly decreased by DEN treatment (*p* < 0.0001), which were recovered by additional TSAHC treatment (*p* = 0.0453), suggesting NK cell activity appeared to be associated with TM4SF5 function/activity-dependent carcinogenesis (Fig. 4E). That is, perforin expression from intrahepatic NK cells appeared to correlate with TM4SF5 function and activity during carcinogenesis, since TM4SF5 inhibition using TSAHC could lead to reduction of NK cells number or function for NK cell exhaustion-like phenotypes.

### TM4SF5 in hepatocytes modulated molecules stimulatory for NK cell surveillance

**Fig. 5:**
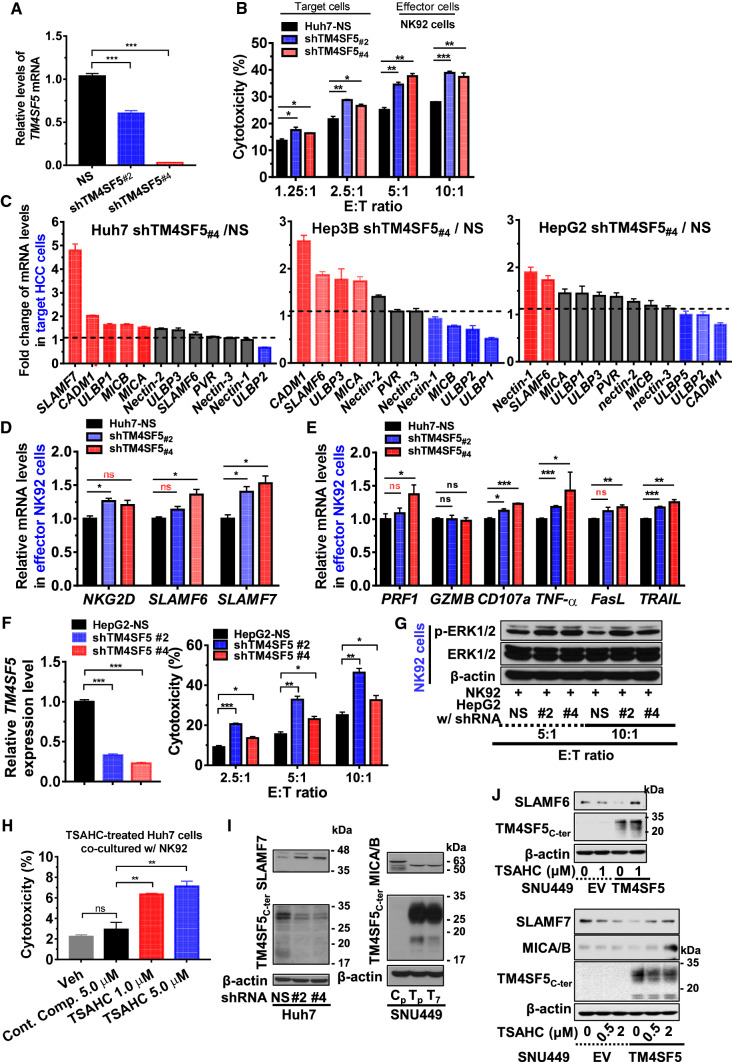
Immune evasion from natural killer cell cytotoxicity via TM4SF5 expression in hepatocytes. **A**–**C** Huh7 (**A**, **B**), Hep3B, and HepG2 cells with endogenous TM4SF5 expression (**C**) were infected with lentivirus for non-specific sequence (NS) or shTM4SF5 (targeting sequences #2 and #4 of *TM4SF5*) before expression level analysis by qRT-PCR for *TM4SF5* or ligands for natural killer cell (NK) cell stimulation (**A**, **C**) or before a NK cell cytotoxicity assay using a co-culture system [at different ratios (E:T) of target (T) Huh7 hepatocytes and effector (E) NK92 cells] for 4 h (**B**). **D**–**G** Huh7 (**D**, **E**) or HepG2 (**F**, **G**) target (T) cells with or without TM4SF5 suppression (**A**, **F**, left) were co-cultured with NK92 effector (**E**) cells for 4 h at an E:T ratio of 10:1 (**D**, **E**) or the indicated ratios (**F**, **G**) for NK92 cytotoxicity analysis (**F**, right), qRT-PCR (**D**–**F**, left), or whole-cell extract preparation prior to immunoblot analysis (**G**). **H** For NK92 cytotoxicity analysis, Huh7 target cells and NK92 effector cells were co-cultured for 6 h (E:T = 10:1), and 4′-(*p*-toluenesulfonylamido)-4-hydroxychalcone (TSAHC) was administered at the indicated concentrations. Statistical significance is indicated by *, **, ***, or ****, which represent a *p*-value ≤ 0.05, 0.01, 0.001, or 0.0001, respectively, and ns indicates no significance, but red-highlighted ns indicates 0.05 ≤ *p*-values ≤ 0.10. *p-*values were calculated by one-way analysis of variance (ANOVA) or one-tailed and unpaired Mann–Whitney *U* tests. **I**, **J** Whole-cell extracts from hepatocytes with different TM4SF5 expression levels (**I**) and from hepatocytes transiently transfected with empty vector (EV) or TM4SF5 plasmids with or without TSAHC treatment at the indicated concentrations for 24 h (**J**) were prepared for immunoblot analysis of the indicated molecules. MICA/B blot from stable control SNU449C_p_ cells showed multiple bands, although stably TM4SF5-expressing SNU449T_p_ or T_7_ cells and other transiently transfected SNU449 cell lines showed a single band. Data shown represent three independent experiments

We next examined how the NK cell cytotoxicity could depend on the TM4SF5 expression and/or activity in hepatocytes. NK92 (effector) cells were co-cultured with hepatocytes (target cancer cells) at different ratios before measurement of lysis of target cells. Endogenous TM4SF5 in Huh7 cells were suppressed (Fig. 5A), before the co-cultures. Cytotoxicity of NK92 cells following the co-cultures increased when they targeted TM4SF5-suppressed cells, compared to non-suppressed target cells (Fig. 5B). Furthermore, we examined membrane proteins (or ligands) on the target hepatocytes, which could be involved in the regulation of NK cell cytotoxicity. qRT-PCR data from three different target cells, including Huh7, Hep3B, and HepG2, showed increased mRNA levels of co-stimulatory ligands on the target hepatocytes with suppression of endogenous TM4SF5, compared with those of non-suppressed (NS) target hepatocytes (Fig. 5C, red-highlighted). They include *SLAMF6* [signaling lymphocytic activation molecule (SLAM) family member 6], *SLAMF7* (SLAM family member 7), *MICA,* (major histocompatibility complex (MHC) I-related chain A), *MICB* (MHC class I related protein B), *CADM1* (Cell adhesion molecule 1), *ULBP1/2* (UL16-binding protein), and others.

Next, we investigated the TM4SF5-mediated effects on ligand levels and NK cell cytotoxicity after the co-culturing of NK92 effector cells with TM4SF5-expressing or –suppressed target hepatocytes. Analysis of cognate receptor expression of the NK92 cells after co-culture with TM4SF5-suppressed target cells showed increased mRNA levels of natural killer receptor group 2 member D (*NKG2D*, stimulatory receptor for MICA/B and ULBP1/3 ligands), *SLAMF6*, and *SLAMF7* (Fig. 5D). Consistently, suppression of TM4SF5 in the target cells resulted in increased NK92 cell cytotoxicity via elevated mRNA levels of *PRF1*, *CD107a*, *TNF-a*, *FasL*, and *TRAIL* in the NK92 cells (Fig. 5E). Co-culturing of NK92 cells with TM4SF5-suppressed target HepG2 hepatocytes (another target cell line) also resulted in increased cytotoxicity of the NK92 cells (Fig. 5F). Following co-culture with TM4SF5-supppressed target cells, NK92 cells showed higher ERK1/2 activity (Fig. 5G), which is known to be associated with the upregulation of NK cell stimulatory receptors [32]. Consistently, when the target hepatocytes were treated with the TM4SF5 inhibitor, TSAHC, the NK92 cell cytotoxicity increased in a dose-dependent manner (Fig. 5H). Additionally, we found that TM4SF5 expression levels in the target cancer cells showed an inverse relationship with the stimulatory ligand levels for NK cell cytotoxicity (Fig. 5I, left and middle). The levels of stimulatory ligands in TM4SF5-positive hepatocytes were increased or highly maintained after TSAHC treatment, whereas the levels appeared to decrease in TM4SF5-negative cells with TSAHC treatment (Fig. 5I, right).

### TM4SF5 inhibitor abolished TM4SF5/STAT3-mediated precancerous and cancerous phenotypes

**Fig. 6:**
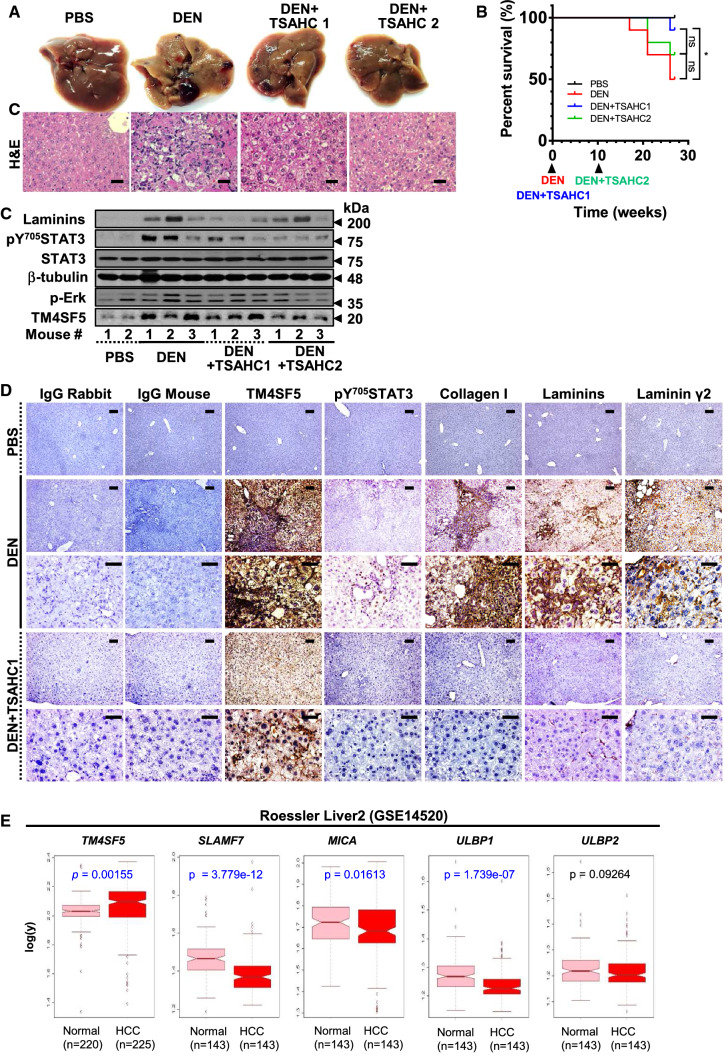
Abolishment of diethylnitrosamine-mediated precancerous and cancerous phenotypes by TM4SF5 inhibition. **A**–**D** Five-week-old BALB/c mice (*n* ≥ 5) were treated with diethylnitrosamine (DEN) for 27 weeks with or without intraperitoneal (IP) injection of DMSO as the vehicle control or 4′-(*p*-toluenesulfonylamido)-4-hydroxychalcone (TSAHC), as explained in the Materials and Methods section. TSAHC was administered either at the beginning of DEN treatment (DEN + TSAHC1) or 10 weeks after DEN treatment (DEN + TSAHC2). Liver tissues were imaged and processed for hematoxylin and eosin staining (**A**). Survival rates of the mice in each experimental group were graphed (**B**). Whole-tissue extracts were also prepared and processed for immunoblot analysis (**C**), or tissues were processed for immunohistochemical analysis of the indicated molecules (**D**). Immunoglobulins (IgG) from rabbit or mouse were used for the negative control stains without the primary antibodies. *p*-values were calculated by Gehan–Breslow–Wilcoxon test for *p = 0.0435. *p*-value > 0.05 was considered statistically insignificant (ns; *p* = 0.3679). Data shown represent three independent experiments. **E** Gene expression profiles of liver cirrhosis [normal (Nor, *n* = 220) vs. HCC (*n* = 225)] from GSE14520 were analyzed and showed HCC with significantly increased *TM4SF5* (*p* < 0.0001), and among the samples showing the increased *TM4SF5* expressing in HCC (*n* = 143) *SLAMF7* (*p* < 0.0001), *MICA* (*p* = 0.0161), and *ULBP1* (*p* < 0.0001) were significantly decreased in HCC, compared with normal counterparts. Meanwhile, *ULBP2* was insignificantly decreased (*p* = 0.0926). *p-*values were calculated by the unpaired, two-tailed Student’s *t* tests

Because we showed that hepatic fibrosis/cirrhosis and cancer involves TM4SF5 (Fig. 2B, C), blockade of TM4SF5 function/activity may be a beneficial strategy for the treatment of TM4SF5-dependent liver diseases. Anti-TM4SF5 compound, TSAHC, was used to block DEN-induced carcinogenesis either at the beginning (DEN + TSAHC1) or during DEN treatment (DEN + TSAHC2). DEN treatment caused cancerous liver nodules, which were abolished by TSAHC treatment (Fig. 6A). Particularly, earlier TSAHC treatment (DEN + TSAHC1) led to increased survival and reduced pY^705^STAT3 and laminin expression levels, whereas later TSAHC treatment (DEN + TSAHC2) did not reduce laminin expression, indicating that earlier TM4SF5 inhibition might be more effective to block the carcinogenesis (Fig. 6B, C). Consistent with these results, decreased laminin expression was evident in mice with earlier TSAHC treatment, but not in those with later TSAHC treatment, leading to a greater survival rate (Fig. 6B). These observations suggest that laminins play an important role in DEN-mediated hepatic carcinogenesis (Fig. 6C, D). Furthermore, public GEO data (GSE14520) analysis showed that *TM4SF5* expression was highly elevated in HCC patients (*p* < 0.0001), and among samples those showing increased *TM4SF5* expression in HCC, *SLAMF7* (*p* < 0.0001), *MICA* (*p* = 0.0161), *ULBP1* (*p* < 0.0001), and *ULBP2* (*p* = 0.0926) were oppositely decreased (Fig. 6E). These observations thus suggest that increased TM4SF5 expression in HCC samples over control counterparts could be linked to decreased expressions of stimulatory NK cell ligand/receptors. Taken together, these results suggest that blockade of TM4SF5 function/activity inhibits liver carcinogenesis, possibly by abolishing TM4SF5-mediated STAT3 activity, the expression of ECM factors, and improvement of NK cell cytotoxicity.

## Discussion

**Fig. 7:**
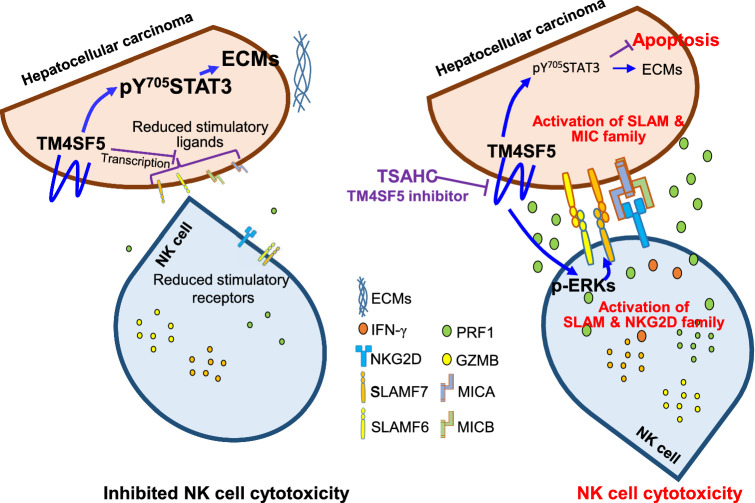
Working models for TM4SF5-mediated suppression of NK cell surveillance. (Left) Highly expressed TM4SF5 in liver cancer cells triggers intracellular signaling for the downregulation of surface ligand-related factors at the transcriptional level, which leads to inhibited NK cell cytotoxicity [i.e., immune evasion from natural killer (NK) cells], in addition to enhanced TM4SF5-pY^705^STAT3 signaling and extracellular matrix production for the promotion of precancerous (fibrotic/cirrhotic) and cancerous phenotypes. (Right) Inhibition of TM4SF5 using anti-TM4SF5 compound, 4′-(*p*-toluenesulfonylamido)-4-hydroxychalcone (TSAHC), upregulates the immune checkpoints between NK cells and hepatocytes, including SLAM, NKG2D, and MICA/B member receptors and ligands, which leads to the stimulation of NK cell cytotoxic receptors and enhanced NK cell cytotoxicity. Activation of NK cell cytotoxicity upon TM4SF5 inhibition by TSAHC treatment appeared to cause (1) inactive STAT3-mediated activation of ligands in hepatocytes stimulatory for NK cell cytotoxicity [54] and (2) ERK activation-mediated increases in stimulatory receptors in NK cells neighbored with TM4SF5-inhibited hepatocytes (Fig. 5G). TM4SF5-mediated STAT3 activation and extracellular matrix production are blocked by TSAHC treatment, which leads to NK cell cytotoxic activity as a potential immunotherapy for TM4SF5-positive liver cancer

This study provides evidence that TM4SF5-mediated STAT3 signaling promoted collagen I and laminin expression, which were involved in hepatic fibrosis/cirrhosis and eventual carcinogenesis in *TM4SF5*-transgenic and chemically induced mouse models. Furthermore, inhibition of TM4SF5 expression or activity and thereby of STAT3 activity abolished ECM production and cancerous phenotypes in the mice livers. DEN-mediated hepatic carcinogenesis was abolished by *Tm4sf5* gene knockout or TM4SF5 protein inhibition, resulting in an increased population and thereby cytotoxic activity of NK cells. Meanwhile, during blockade of tumorigenesis via the TM4SF5 inhibition but not via TM4SF5-deficiency, Th and/or T cells might play roles in immune surveillance. Suppression or inhibition of TM4SF5 in target hepatocytes (cancer cells) increased the mRNA levels of stimulatory ligands in hepatocytes and cognate receptors in NK cells and also led to enhanced NK cell surveillance (Fig. 7). Therefore, TM4SF5 may be targeted using an anti-TM4SF5 reagent, such as a small compound TSAHC [25], or its antibody [19] as a potential NK cell-related immunotherapy for HCC, in addition to immunotherapies against PD-1, PD-L1, or CTLA-1 [12]. Indeed, we have reported by mutation study and molecular modeling that TSAHC binding to TM4SF5 occurs at a region of the long extracellular loop (W124 to E129 of LEL) caused induced fit changes, leading to inhibitions of protein–protein interaction, l-arginine binding, and its downstream signaling activity [26]. TSAHC is quite specific for TM4SF5 at 0.3–5 μM, since TM4SF5-negative hepatocytes were not responsive to its treatment [25, 33, 34].

On the cell surface, there are certain domains in which specific membrane proteins and receptors are enriched to transduce specific intracellular signaling pathways. Such domains include focal adhesions, lipid rafts/caveolae, and tetraspanin-enriched microdomains (TERMs). Similar to focal adhesions with integrins that are engaged with the ECM and lipid rafts/caveolae that are enriched with glycosylphosphatidylinositol-linked proteins, TERMs are enriched with tetraspanins for massive protein–protein complexes that include tetraspanins, integrins, and growth factor receptors [35]. TM4SF5, a member of the transmembrane 4 L six family or L6 four transmembrane superfamily also forms TM4SF5-enriched microdomains (T_5_ERMs) [15]. Thus, TM4SF5 at T_5_ERMs may function differentially depending on cellular needs, via changes in binding partners or signaling activities/pathways to transduce diverse intracellular signaling pathways in a spatio-temporal manner [36]. TM4SF5 expression in hepatocytes promotes phosphorylations of FAK [37], c-SRC [20], STAT3 [21, 38], and mTOR/S6K1 [26]. TM4SF5 also binds EGFR [39], integrin α5 [38, 39], CD133 [34], CD151 [40], CD44 [41], and mTOR [26] membrane receptors or proteins. Interaction of TM4SF5 with integrin α5 induces VEGF secretion for hepatic angiogenesis via enhanced proliferation and migration of endothelial cells [38]. In addition, we have observed that TM4SF5 is involved in bidirectional crosstalks between hepatocytes and macrophages during development of NASH and is induced in differentiation/activation of macrophages or T cells (data not shown).

TM4SF5-mediated protein–protein complex formation at the T_5_ERMs may serve as a signaling hub of diverse signaling pathways for gene expression or suppression at the transcriptional level. It may also modulate the expression levels of its binding partners via subcellular trafficking or compartmentalization prior to degradation or stabilization. In lung epithelial cells, TM4SF5 expression leads to suppression of ZEB2, which in turn increases alternative splicing factors for CD44 isoforms (from the standard CD44 form to CD44_v8-10_ variant form) during idiopathic pulmonary fibrosis [42]. Furthermore, TM4SF5 expression in hepatocytes leads to the downregulation of *E-cadherin* mRNA levels, thus promoting the loss of contact inhibition via epithelial–mesenchymal transition [17]. Here, we have shown that TM4SF5 decreased the mRNA levels of NK cell stimulatory receptors, such as *NKG2D* (receptor of MICA/B and ULBP1/3 ligands), *SLAMF6*, and *SLAMF7*, and of hepatocyte cognate ligands, via immunoblot experiments and public GEO data analyses. Therefore, it can be likely that TM4SF5 transcriptionally regulated gene expression and was involved in tumor progression. Here, we also found that ERK1/2 phosphorylation increased in NK cells after co-culturing with TM4SF5-suppressed target hepatocytes (Fig. 5G). Consistently, NK cell activation via the expression/activation of stimulatory receptors including those of the self-specific SLAM family for lymphocyte activation [43] and via lytic granule polarization and secretion [44], requires ERKs activation [32].

In addition, TM4SF5-mediated STAT3 activation appeared involved in excessive ECM production and regulation of stimulatory ligands and receptors levels for NK cell cytotoxicity. It is well known that collagen I expression is important for liver fibrosis [45]. Meanwhile, our present study shows that active STAT3 correlated with laminin and laminin γ2 expression, in addition to collagen I, and also with subsequent hepatic carcinogenesis. Active STAT3 in hepatic inflammatory environment has been shown to induce ECM expressions by binding to their promoter regions [46]. Considerable immunostaining for laminin γ2 is shown in the cytoplasm of hepatic carcinoma cells [47] and laminin γ2 is suggested as a novel serum biomarker for HCC [48]. Indeed, TM4SF5 has been previously reported to be related to liver cancer progression, together with cell markers CD133^+^, CD44^+(bound to TM4SF5)^/CD24^−^/ALDH^+^/Bmi^+^, and protein tyrosine phosphatase receptor type F (PTPRF^−^) [34, 41], in addition to laminin γ2^+^, AFP^+^, MCP-1^+^, CD34^+^, and FUCA^+^ (AFU) shown in this study. TM4SF5 interacts with IL6R, leading to STAT3 activation independent of IL6 in hepatic cancer cells [21]. Because c-SRC is upstream of STAT3 [49] and TM4SF5 directly binds and activates c-SRC [20], TM4SF5 may therefore activate STAT3 in a ligand-independent manner. Indeed, IL6 activates STAT3 during HCC growth [50]. However, small in-frame deletions around the gp130 binding site for IL6 [51] and disruption of negative regulators of STAT3 such as SOCS3 and SHP1/2 [52, 53] are involved in ligand-independent STAT3 activations. Recently we reported that TM4SF5 activates STAT3 for the pathological progression to NASH-associated with fibrosis via hepatic inflammation-concomitant SOCS1/3 down-regulation [24]. Here, a negative correlation was shown between *TM4SF5* and *SOCS1* expression in HCC patient groups. Therefore, TM4SF5 at T_5_ERMs can promote STAT3 activity during the progression toward fibrotic and cancerous liver phenotypes. The blockade of TM4SF5-dependent STAT3 activity and liver carcinogenesis by TSAHC treatment appeared further to be correlated with improved NK cell cytotoxicity.

The inhibition of TM4SF5 in (target) hepatocytes appeared to activate NK cell surveillance via different signaling pathways; (1) inactivation of STAT3 activity leading to apoptosis directly and activation/induction of stimulatory ligands in hepatocytes for NK cell surveillance indirectly, and (2) activation of ERKs in NK cells for increased SLAM and NKG2D receptor families for improvement of NK cell cytotoxicity. Consistently, STAT3-blocked (or inhibited) hepatocellular carcinoma cells showed upregulated NKG2D ligands (i.e., MICA/B and ULBPs), and thereby cytotoxicity of NK cells treated with supernatant from STAT3-blocked hepatocytes was augmented with a concomitant elevation of molecules associated with NK cytolysis [54, 55]. Interestingly, we have not found any cytokines, chemokines, growth factors, or extracellular matrix that could induce TM4SF5 in NK92 cells upon their treatment alone or in combinations (i.e., tested with approximately 40–50 different factors, data not shown). Indeed, here TM4SF5 knock-out and inhibition (by TSAHC) led to less significant tumor formations in xenografts. However, NK cell cytotoxicity appeared functional upon TM4SF5 inhibition in vitro cells and in vivo animals but not upon *Tm4sf5* gene knock-out. Although Th and T cells were shown to be involved in the TM4SF5 inhibition-mediated blockade of tumorigenesis, those immune cells appeared not to be critical for the DEN-mediated tumorigenesis in WT mice, compared to significantly less tumorigenesis in KO mice. Thus, we may speculate that in vivo complicated system without *Tm4sf5* gene and related pathway may adopt other immune system (such as Th and/or T cells) or cytotoxic pathway (no survival/growth due to no TM4SF5-STAT3 activation). Presumably, conditional *Tm4sf5* KO may be informative on whether the compensatory mechanisms may be the development of the immune system without TM4SF5 involvement. By the way, TM4SF5 in hepatocytes can still be targeted by an anti-TM4SF5 reagent, including TSAHC [25] and its antibody [19].

Altogether, we provide evidence that TM4SF5 plays an important role in the pathological progression of liver carcinogenesis via TM4SF5-mediated STAT3 signaling for ECM production. Concomitantly, TM4SF5 mediates the downregulation of stimulatory membrane ligands on target hepatocytes that stimulate NK cell cytotoxicity, leading to NK cell immune exhaustion-like phenotypes including reduction of NK cells number or function upon TSAHC treatment. Thus, TM4SF5 (as a molecule to suppress NK surveillance) is a promising target for the treatment of advanced liver diseases.

## Data Availability

Upon written requests to the corresponding author, the data and materials can be available.
